# An overview of progress towards implementation of solid waste management policies in Dhaka, Bangladesh

**DOI:** 10.1016/j.heliyon.2022.e08918

**Published:** 2022-02-09

**Authors:** Delufa Tuz Jerin, Hasna Hena Sara, Marzuka Ahmad Radia, Prianka Sultana Hema, Shahriar Hasan, Salma Akter Urme, Camilla Audia, Md. Tanvir Hasan, Zahidul Quayyum

**Affiliations:** a BRAC University, Bangladesh; b Kings College, London, UK

**Keywords:** Public policy, 3R, Solid waste management, Bangladesh

## Abstract

**Objectives:**

Considering the increased solid waste generation and its management, this paper aims at reviewing and identifying the gaps and challenges in implementing the existing solid waste management relevant policies, strategies and action plans in Bangladesh for providing further strategic recommendations to establish a sustainable waste management system.

**Methods:**

This study adopted a multi-method approach by reviewing 24 policy/strategy documents; implementation gap analysis with extensive desk review and data obtained from the qualitative approach and co-production workshop. It allowed this study to capture the multidimensional and comprehensive scenario of waste management in Dhaka city.

**Results:**

Bangladesh has undergone reforms in solid waste management since 1983 with the adoption of the Dhaka City Corporation Ordinance and the enactment of the National 3R Strategy in 2010. With few exceptions, the overall waste management system cannot be defined as an effective comprehensive waste reduction, recycling and disposal mechanism based on global standards. Study found that several action plans have been introduced yet not all of those have proper implementation like adhering the waste segregation and recycling practices from households to landfill level. Lack of monitoring and coordination among the existing policy implementing agencies have emerged as significant concerns in Bangladesh.

**Conclusion:**

Challenges in the implementation of pragmatic and improved policies and strategies should be addressed.

## 1 Introduction

Globally, rapid urbanization and industrialization over the past decades have led to better socio-economic conditions and improved consumption patterns among city populations [1, 2, 3]. Such growth in prosperity and abundance of resources bring in high per capita waste generation [4]. A greater volume of inexorable waste is generated as a byproduct of household consumption. The world generated 0.68 billion tonnes of municipal solid waste (MSW)^1^ (0.68 kg per capita per day) in 2002 which amplified to 2.01 billion tonnes annually (0.74 kg per capita per day) in 2018 [4, 5]. The global MSW generation is expected to grow 3.40 billion tonnes annually by 2050 [4]. Managing such a large volume of MSW in an environmentally acceptable way is a major challenge in urban areas throughout the world, particularly in rapidly growing cities of developing countries [6, 7]. According to Waste Atlas, almost 30 percent of waste generated globally remains uncollected [8].

In Bangladesh, the volume of waste generated in 1970 was 11,00,000 tonnes which amplified to 1,47,78,497 tonnes in 2012 with an annual increase of 1,34,300 tonnes [4, 9, 10]. The urban areas generated 5,200,919 tonnes per year in 2014 (0.35 ​kg per capita per day) [10]. According to the most recent data, average per capita MSW production in different municipal areas ranges from 0.2 to 0.56 kg/cap/day [11]. In 2016–2017, Dhaka, the capital city of Bangladesh generated 6448.373 tonnes of MSW per day (0.57 per capita per day) [12].

Approximately half of the waste generated in Dhaka city is collected properly by City Corporation and almost 40–60% of the waste remains uncollected and is not safely disposed of where the waste has 80% organic content [11, 13]. Dhaka city already facing several challenges due to sky-scraping waste volumes and it is predicted that the total urban population will reach 78.44 million by 2025 and the rate of waste generation is projected to increase to 220 kg per capita per year in 2025 [14]. In the case of waste disposal in landfill, the amount of land area is also scarce compared to waste generation. These extenuating factors, such as scarcity of land and a lack of technical skills to deal with massive volumes of waste, as well as a lack of definitive legislative policies, exacerbate the situation [7]. This poor municipal solid waste management system becomes a challenge for the city and it is also a crucial concern for the protection of public health, safety, and the environment while also impacting the economic development and quality of life [3, 7, 15]. Hence, in the above context, it is very important to ensure safe and proper disposal of the generated solid waste to maintain the serenity and the efficiency of the city. However, disposal of solid waste is a serious problem because if burnt it can lead to an increase in air pollutants, and if openly dumped can lead to soil and water contamination of the surrounding regions.

Similar to other developing nations, Bangladesh follows conventional municipal solid waste management. The common practice here includes some obsolete waste management practices consisting of irregular waste collection and crude open dumping that apparently causes air, soil, and water pollution [16]. MSW management in Bangladesh incurs a number of problems including resource constraints with limited financing, technical difficulties, lack of public awareness, lack of coordination between different government departments and public and private sectors [7,9,17, 18, 19]. As MSW management falls completely under the local government's jurisdiction, central government ministries have little jurisdiction over waste collection, treatment, and disposal services. However, different ministries such as The Ministry of Environment, Ministry of Planning, Local Government and Rural Development department, etc. play a significant role in policy formulation and policy decisions regarding sustainable solid waste management.

In the past few decades, many different policies and action plans have been formulated and experimented by the central and local government (City corporations and Municipalities) of Bangladesh to improve the MSW management system in urban areas but due to lack of awareness of city dwellers and poor implementation of policies and actions plans major improvement has not been observed. The situation is getting worse gradually and making the current MSW management system ineffective. Though several studies have been conducted in the Bangladesh context, it is important to review the existing policies, rules, regulations, and legal frameworks and revise accordingly. Thus this paper aims at identifying the gaps and challenges in existing solid waste management policies and action plans in Bangladesh. It also documents the implementation challenges and recommends strategies to improve the situation.

## 2 Materials and method

BRAC James P Grant School of Public Health (JPGSPH), BRAC University conducted a study on MSW management in Dhaka, the capital city of Bangladesh from July 2019 to January 2020 under the project Pathways to Equitable Healthy Cities (Pathways). The study adopted a combination of research methods (desk review, secondary data collation and analysis, and primary data collection and analysis) to obtain a deeper understanding of the different issues of municipal solid waste management in Dhaka city.

This paper is based on a part of the above-mentioned waste management-focused study. It particularly focused on the existing policy reviews and implementation gap analysis to summarize the findings from a qualitative study, co-production workshop, and an extensive literature review, which assembled a comprehensive list of waste management policies, strategies, and action plans. The definitions of ‘‘policy’’ and ‘‘action plan’’ vary broadly among the national documents. For the purpose of this paper, a broad definition of the policy was used, and all national documents including national objectives, strategies, and guidelines for action in the waste management domain were included, irrespective of their title (e.g., ‘‘plan’’ versus ‘‘policy’’ versus ‘‘strategy’’). We have searched the government portals and website of the different ministries involved in planning and implementing environmental or waste-management related policies. We have also visited the city corporation websites to collect all policy-related documents. All these documents were screened based on the following inclusion criteria: a) the policy mentioned activities related to managing waste (solid, industrial, biomedical, agricultural); b) the policy was publicly available and published between 1 January 1972 and 1 January 2020. After extracting all policies, strategies, action plans and ordinances related to waste management, we have reviewed all those documents. After screening finally, twenty-four documents of national policies, ordinances and strategies were considered to be relevant to address the research objectives. Then a brief policy review matrix is developed based on 24 policy documents.

In addition to the policy review, this paper utilized the data obtained from field observation using two types of observation checklists, one for the Secondary Transfer Stations (STSs) and another one for the 2 landfills; Key Informant Interviews (KIIs), and finally organized a co-production workshop on solid waste management in Dhaka city in September, 2020. Also, two separate observation checklist (which includes infrastructure, logistics, condition and management practices) was used to assess the condition of 81 STSs and two landfills. Snowball sampling method was used to recruit the respondents for KIIs, where the respondents were asked a number of questions including waste management-related policy gaps and challenges of those policy implementations for the further recommendations. The KII respondents included government and non-government professionals who are engaged in policy formulation, implementing several policies and programmes related to waste management and researchers from academia engaged in research on solid waste management.

The study also utilized the findings obtained from the online co-production workshop titled “Solid Waste Management in Dhaka city: Establishing Practices and Conduits towards a Sustainable Waste Culture”, organized by James P Grant School of Public Health, BRAC University on 17 September, 2020. In the workshop, the research team shared preliminary findings of the desk review, observation checklists and KIIs with diverse stakeholders from government officials, researchers and academics and different NGOs. Later the workshop attendees were asked to share their thoughts on the preliminary findings and recommend strategies that can be adopted to fill in the policy gaps and improve the MSW management scenario of Dhaka city.

Qualitative data were analyzed using Nvivo software. By assigning codes to emerging trends and systemic themes, and keeping in mind the outcomes of the co-production workshop, we examined and cross-checked the implementation gaps and limitations of each policy or strategy. Thus, this multi-method approach allowed us to capture multidimensional realities and a comprehensive scenario. After that, the recommendations were provided based on amalgamating and integrating the findings from the assessment of STSs and landfills, co-production workshop, qualitative data analysis, desk review, and the review of policies.

### 2.1 Ethics approval

This study has received ethical approval from the Institutional Review Board (IRB) of BRAC James P Grant School of Public Health (Protocol No: 2019-012-IR), BRAC University.

## 3 Results

The findings of this study include two major sections of which section one contains the policy matrix with the summary of all the available policies and strategies along with the action plans regarding those. Section two is a review of the implementations and gaps of these existing policies in a thematic way under 5 other sub-sections, i) waste generation; ii)waste collection and transportation; iii) waste disposal; iv) waste recycling and treatment & v) monitoring, regulation, and governance. The triangulation of methods helps us to validate the research finding by utilizing data obtained from different methodological approaches as discussed under the methods section [20].

### 3.1 Policy matrix

To prepare the policy matrix, list of the documents were considered are: Constitution of Bangladesh (CoB) 1972, The Environmental Pollution Control Ordinance 1977, Dhaka City Corporation Ordinance 1983, National Environment Policy 1992, National Environmental Management Action Plan (NEMAP) 1995, Environment Conservation Rule 1997, National Policy for Water Supply and Sanitation 1998, National Agriculture Policy 1999, Private Sector Infrastructure Guideline 2004, Dhaka Declaration on Waste Management by SAARC countries during 10–12, October 2004, Dhaka Environment Management Plant 2005, Poverty Reduction Strategy Paper (PRSP) 2005, Private Sector Housing Development Guideline 2005, Solid Waste Management Action Plan for Eight Secondary Towns in Bangladesh 2005, National Renewable Energy Policy 2008, Biomedical Waste Management Rules 2008, Local Government (City Corporations) Act 2009, National 3R Strategy 2010, The Bangladesh Environment Conservation Act 2010, National Urban Sector Policy 2011, Directions from the High Court to stop dumping waste in the Buriganga River, Dhaka 2011, National Strategy for Water Supply and Sanitation 2014, Dhaka Structure Plan 2016, Seventh Five-year plan (FY2016-2020). These documents are considered for further analysis of the study (see Table 1).

**Table 1:** Framework of relevant waste management policy directives.

National policies, ordinances & strategies	Waste management relevant focus areas	Necessary actions for Implementation
1. Constitution of Bangladesh (CoB) 1972 [51]	The CoB in Article 18A focuses on the improvement of the environment and natural resources for future generations, a positive aspect of the fifteenth amendment (Article 18A). • **Implementing partners:** Government of the People's Republic of Bangladesh	1. Protect and improve the environment by preserving and safeguarding the natural resources, bio-diversity, wetlands, forests and wildlife for the present and future citizens
2. The Environmental Pollution Control Ordinance (1977) [52]	This ordinance includes the following action points: 1. Makes provision for the constitution of the Environment Pollution Control Board consisting of representatives of various public bodies. 2. Allows the Board to formulate policies for the control, prevention and abatement of pollution of the environment • **Implementing partners:** Government of the People's Republic of Bangladesh - Department of Environment (DoE), Ministry of Environment and Forest (MoEF)	1. The board director may allocate a person/organization to construct, modify, extend or alter any disposal system including waste disposal system 2. Provision of information by the owner/occupier of a specific building/land relating to wastes, sewerage system or treatment works of that building/land 3. All persons/organizations must grant permission to enter, inspect and search their owned/occupied land or building and to inspect and test any wastes, air, water, materials of the disposal system
3. Dhaka City Corporation Ordinance, 1983 [53]	This ordinance was formed to consolidate and amend the law relating to the municipal administration of Dhaka City. The ordinance states regarding the MSW management that 1. Dhaka City Corporation (DCC) is responsible for: a. Removal, collection and disposal of wastes, b. Cleaning drainage, cleansing of streets 2. Building occupiers are responsible for removing refuse from residing buildings. • **Implementing partners:** Government of the People's Republic of Bangladesh, local government (DCC), NGOs and INGOs and private companies	DCC actions include: 1. Providing public dustbins and receptacles within the City corporation area 2. Public notification of refuse accumulation by building occupiers in provided dustbins 3. Employing manpower for MSW collection and refusal of MSW 4. Making adequate arrangements for the refuse removal from all public streets, public latrines, urinals, drains and all buildings and lands within the area. Civil society actions include: 1. Carrying/disposing of their waste in the waste receptacles installed by DCC either by themselves or through contacting an NGO/CBO/Private company
4. National Environment Policy 1992	This policy includes the following objectives to ensure environmental sustainability: 1. Aim to provide protection and sustainable management of the environment; 2. Maintain the ecological balance and overall development through protection and improvement of the environment; 3. Identify and regulate polluting and environmentally degrading activities; 4. Ensure environmentally sound development; 5. Ensure sustainable and environmentally sound use of all-natural resources; and 6. Actively remain associated with all international environmental initiatives • **Implementing partners:** Ministry of Environment and Forest, A National Environment Committee chaired by the Head of Government	1. Establish "waste permit/consent order" system in the industrial sector; 2. Encourage recycling 3. Take appropriate measures on an emergency basis to remove and properly dispose of garbage & waste of oil/oil products from ships at Chittagong and Mongla port.
5. National Environmental Management Action Plan (NEMAP), 1995 [54]	This action plan focuses on sanitation, solid waste management, water supply and environmental awareness among the citizen to fulfill the following objectives: 1. Strengthen local government institutional capacity for integrated planning of the policies 2. Create better housing facilities for the urban population including poor and middle class 3. Properly manage the urban sewage and disposal of solid waste/household waste • **Implementing partners:** Ministry of Environment and Forest (MoEF), Local Governments and NGOs	1. Proper management of the urban sewerage and disposal of urban solid waste/household waste 2. Properly handle sewerage and garbage disposal issues and ensure better garbage disposal and sewerage facilities 3. Develop garbage disposal and sewerage treatment capability by recycling and economic use of wastes.
6. Environment Conservation Rule, 1997 [55]	The Environment Conservation Rule focuses on the following: 1. Specifies the inclusion of the following: i. Pollution Under Control Certificate, ii. Fees for Environmental Clearance Certificate and other services etc. 2. To declare any area as an Ecologically Critical Area these factors need to be considered by the Government: Human habitat, Ancient monument, Archeological site, Forest sanctuary, National park, Game reserve, Wild animals' habitat, Wetland, Mangrove, Forest area, Bio-diversity of the relevant area.	1. Determine standards for Waste from Industrial Units. 2. Enforce determined standards in a more stringent manner if considered necessary in view of the environmental conditions of the particular situation. 3. Determine the standards of odors 4. Determine the Standards for Sewage Discharge 5. Categorize landfill activity with industrial, household and commercial waste as a red category activity 6. To undertake any landfill project, consider environmental impact assessment and obtain no-objection certificates
7. National Policy for Water Supply and Sanitation, 1998 [56]	This policy includes the following provisions for waste management: 1. Special emphasis on the private sector and NGO participation in urban water supply and sanitation 2. Clearance for local government to transfer collection, removal and management of solid waste to the private sector where feasible 3. Promotion of maximum waste recycling and use of organic waste materials for compost and biogas production in the private sector **Implementing partners:** Government of the People's Republic of Bangladesh, Local Government and Rural Development, private sector and NGOs	1. Feasible collection, removal and management of solid waste involving the private sectors. 2. Recycle maximum amount of waste 3. Promote the use of organic waste materials for compost and biogas production.
8. National Agriculture Policy 1999 [57]	The National Agricultural policy includes the following aims: 1. Making the nation self-sufficient in food through increasing production of all crops including cereals 2. Ensuring a dependable food security system for all. 3. Developing processing facilities to reduce wastage of rapidly perishable crops, increase utility and maintain quality of agricultural commodities • **Implementing partners**: Ministry of Agriculture	1. Develop processing facilities to reduce wastage of rapidly perishable crops
9. Private Sector Infrastructure Guideline 2004 [58]	These guidelines include waste management relevant infrastructural guidelines that 1. Promotes the development of infrastructure projects through the private sector. 2. States that infrastructure projects on environmental, industrial and solid waste management may be implemented as private infrastructure projects • **Implementing partners:** Government of the People's Republic of Bangladesh, private infrastructure committee (shall be established under the prime minister's secretariat), project company (which get tender)	1. Private sector firms to submit a solicited/unsolicited proposal to pursue a project 2. The government to identify the codes and standards for the design, construction, operation and maintenance of proposed infrastructure, following the proper sanitation and waste management systems
10. Dhaka Declaration on Waste Management by SAARC countries during 10–12, October 2004 [58]	The Dhaka Declaration on waste management addressed the following: 1. Stop open dumping immediately 2. Replace the open dumping sites with new safe disposal options (controlled landfill sites). 3. encourage NGOs and private companies to establish a. Community-based composting b. Segregation of waste at source, c. Separation, collection and resource recovery from wastes with particular focus on composting • **Implementing partners:** SAARC countries.	1. Promote an effective, efficient, affordable, safe and sustainable waste management system of all the urban/rural settlements 2. Establish a SAARC network on waste management; 3. Incineration of waste; 4. Put the particular focus on composting; 5. Ensure special treatment of hospital waste; 6. Privatize waste collection, disposal and treatment.
11. Dhaka Environment Management Plan 2005 [59]	The Dhaka Environment Management plan included the following initiatives: 1. Ensure sustainable urbanization through decentralized development and a hierarchically structured urban system. 2. Emphasize recycling as a means to reduce solid waste management cost, under urban environmental management dimension • **Implementing partners**: Government of the People's Republic of Bangladesh, local government and rural development and cooperation	1. Promote waste recycling 2. Encourage less landfilling 3. Promote the development of an EMS (Environmental Management System) among industries
12. Poverty Reduction Strategy Paper (PRSP) 2005 [60]	The PRSP emphasizes on: 1. Ensuring environmental balance in all sorts of development activities. 2. Importance of proper waste management and recycling activities, 3. An environment-friendly energy policy and social forestation • **Implementation partners:** Local Government Division, Ministry of Environment and Forest, Ministry of Law Justice and Parliamentary Affairs, Ministry of Local Government and Rural Development and Cooperative, Ministry of Social Welfare, Ministry of Health and Family Welfare, City Corporation, Municipalities, Civil Society Organizations and NGOs	1. Introduce segregation of organic and nonorganic waste at the household level 2. Encourage public-private partnership for MSW management 3. Formulate and implement solid waste management master plan for each municipality, and replicate them in other major cities 4. Popularize private-public partnership in waste removal process in cities 5. Improve waste disposal systems and their management substantially 6. Introduce sanitary land-fill for all solid waste disposal 7. Recycle, reduce and reuse of industrial and other solid waste
13. Private Sector Housing Development Guideline 2005 [61]	The Private Sector Housing Development Guidelines provide assistance to the proper waste management systems in order to ensure waste minimization and waste removal and produce pollution-free goods. • **Implementing partners:** Government of the People's Republic of Bangladesh (Ministry of Industry), private sector, NGOs	Proposes waste recycling, composting and biogas generation space in the housing areas following the development guideline
14. Solid Waste Management Action Plan for Eight Secondary Towns in Bangladesh 2005 [62]	This action plan was devised by Waste Concern and based on the 4 R principle i.e. Reduce, reuse, recycle and recovery the waste. It focused especially on the promotion of converting waste into resource activities. • **Implementing partners**: Local Government Engineering Department	a) Solid Waste Management and Resource Recovery, b) Clinical & Hazardous Waste Management, c) Policy on Waste Management, d) Climate Change & Clean Development Mechanism, e) Industrial Pollution Control
15. National Renewable Energy Policy 2008 [63]	This policy includes directives on energy generation from waste and mentions the following key issues: 1. Sources for biomass gasification-based electricity, such as: rice husk, crop residue, wood, jute stick, animal waste, municipal waste, sugarcane bagasse etc. 2. Defines biogas from mainly animal and municipal wastes as a probable promising renewable energy resource for Bangladesh. **3. Implementing partners:** The Sustainable Energy Development Agency (SEDA), business community, academics and/or representatives from Bangladesh Solar Energy Society, NGOs, financial institutions	1. Promote awareness about biomass and composts among the public 2. Introduce new business models for renewable energy and other clean energy technologies 3. Create market opportunities and start-up business models for sustainable energy technologies in Bangladesh
16. Biomedical Waste Management Rules 2008	This set of rules defines the following activities: 1. Every occupier/institution generating bio-medical waste are responsible to ensure that such waste is handled without any adverse effect to human health and the environment. 2. Every occupier/operator to submit an annual report to the prescribed authority by 31 January, including information about bio-medical waste handled during the preceding year. 3. In case of any accidents at any bio medical waste handling site or during transportation, the authorized person shall report the accident to the prescribed authority forthwith. • **Implementing partners:** The Government of every State and Union Territory, Municipal Corporation, Municipal Boards or Urban Local Bodies	1. Set up a monitoring team for segregation and disposal of a different kinds of medical waste 2. Categorize all medical waste in 10 categories with different handling protocols
17. Local Government (City Corporations) Act 2009	This act holds city corporations responsible for the following activities: a) Proper disposal of waste b) Collection of waste c) Management of waste. • **Implementing partners:** City corporation	1. Ensure proper disposal, collection and management of waste
18. 2010, National 3R Strategy [64]	This strategy aims to change the consumption and production patterns of wastes by: 1. Directing the local government authorities to develop their own action plans through quantifiable targets 2. Encouraging organic waste recycling through composting, bio-gas, and refuse-derived fuel. The goals include: 1. Waste reduction by 20% 2. Reuse and recycling 3. Minimizing waste disposal in open dumps, rivers, flood plains and landfills by at least 20% within 2015. • **Implementing partners:** MOLGRD, MOEF, MOI, MOH	1. Identifying pilot wards of Dhaka city 2. Distributing collection vans and household garbage bins 3. Producing source-separation instruction tools 4. Raise awareness about waste reduction, recycling and reusing through Environmental education & public relation activities 5. Implement source separation; 6. Develop Material Recovery Facility (MRF); 7. Construct compost plant 8. Sell and produce compost
19. The Bangladesh Environment Conservation Act,2010 [65]	This act was amended in 2010 and includes the following waste management relevant issues: 1. Includes many important environmental concerns such as conservation of wetlands, hill cutting, ship breaking, and hazardous waste disposal. 2. The discharge, disposal and dumping of waste can cause adverse environmental effects and some type of wastes can be considered as environmental pollutants 3. It also focuses on the a. Conservation of environment, b. Improvement of the environmental standards, c. Control and mitigation of environmental pollution; • **Implementing partners:** Government of the People's Republic of Bangladesh, NGOs, Private Sector	1. Provide restrictions on the production, import, storage, loading, transportation etc. of • hazardous waste, • anything defined as waste, due to its physical or chemical properties • if contraction with other waste or substances creates toxicity, infection, oxidation, exploration, radioactivity, decay or other harmful environmental effects
20. National Urban Sector Policy, 2011 [66]	This policy addresses the following issues: 1. Promotes sustainable urbanization through decentralized development and a hierarchically structured urban system 2. Reduce solid waste management cost through emphasizing recycling and ensuring government support for the same 3. Focuses on improvement of urban infrastructure quality and technology for maintenance 4. Includes routine maintenance, periodic maintenance, emergency maintenance, and rehabilitation under routine maintenance policy • **Implementing partners:** Government of the People's Republic of Bangladesh, Local Government and Rural Development and Cooperation	1. Introduce user fees for waste disposal, encouraging composting, and formalizing the function of scavengers for MSW management. 2. Ensure investment to replace and repair and maintain infrastructure facilities.
21. Directions from the High Court to stop dumping waste in the Buriganga River, Dhaka, 2011	The High Court of Bangladesh has enforced this law several times to stop illegal waste dumping on the banks of the Buriganga river and protect the river water	1. City authorities to run programmes to create awareness among people on dumping waste into and along the river and put up placards sporting the High Court direction. 2. All sewerage lines connected to the Buriganga and waste treatment lines from industries have to be delinked from discharging liquid wastes into the rivers within a year. 3. Directed the authorities to stop dumping waste into the river and declared their inaction in preventing water pollution illegal and clean up the river and move all the sources of pollution from there. 4. Department of Environment to shut down industries built on the banks of Buriganga without clearance and the industries polluting the river by dumping waste, within the next one month, following June 2020. 3. Ordered the authorities to stop dumping all types of waste to keep the water pollution-free.
22. National Strategy for Water Supply and Sanitation, 2014 [67]	This national strategy on sanitation: 1. Aims to ensure safe and sustainable water supply, sanitation and hygiene services for all 2. Adopts 17 strategies (with strategy 6 focused on MSW management), which are broadly grouped into three themes: a. Increasing water, sanitation, and hygiene (wash) interventions b. Addressing emerging challenges c. Strengthening sector governance • **Implementing partners:** Government of the People's Republic of Bangladesh, Department of Public Health and Engineering, Local Government Division, Local Government Engineering Department, Dhaka Water Supply and Sewerage Authority and NGOs	1. Promote source-level waste segregation 2. Establish community based primary collection and link with secondary collection transportation and final disposal 3. Consider special handling and treatment for hazardous, electronic and medical waste 4. Pursue organic waste recycling through composting, bio-gas and reuse derived fuel 5. Plan sanitary/regional landfills for an urban area/a group of urban areas 6. Design sanitary landfills with the provision of using methane gas as fuel 7. Prevent keeping waste materials on footpaths, roadsides and other public places.
23. Dhaka Structure Plan 2016 [68]	Contains several articles that have a focus on MSW management and aims to 1. Ensure minimized waste generation and create a clean and pleasant living environment 2. Establish waste transfer stations at proper places and prevent public nuisance 3. Ensure greater private sector participation in MSW management 4. Introduce health and hygiene counseling and healthy practices at home and schools 5. Create environmental awareness among people • **Implementing partners:** Local Government Agencies, RAJUK, DOE and NGOs, Ministry of Education, Ministry of Culture	1. Adopt the 3R policy (Reduce, Reuse and Recycle). 2. Consider the necessity of alternative landfill sites as the least preferred option 3. Introduce hierarchy classification to extract the maximum practical benefits from products whilst generating the minimum waste 4. Manage medical waste with care and hygiene. 5. Evolve various initiatives to design and ensure safe recycling of electronic products 6. Select feasible locations of STSs to prevent public nuisance. 7. Consider underground tunnels for dispatching household waste to waste-treatment centers 8. Engage the private sector to initiate ideas to make the waste collection a profitable business 9. Undertake programmes and projects and allocation of budget for projects to promote healthy living 10. Engage in public awareness-raising actions in the form of seminars, workshops, TV advertisements, newspaper supplements, dramas
24. Seventh Five-year plan (FY2016-2020) [69]	The 7^th^ five-year plan includes several action directives that aims the following: 8. To ensure a proper MSW management system in place for good environmental health. 9. To implement the emission, effluent, and waste management strategy. 10. Entrusts the Local Government Division with the following: a) managing all matters related to drinking water; b) developing water supply, c) sanitation, and sewerage facilities in rural and urban areas d) Managing matters related to waste management. 11. To ensure the practice of 3R (Reduce, Reuse & Recycle), following the National 3R Strategy for Waste Management 12. To enforce Solid Waste Management Rules. **Implementing partners:** Government of the People's Republic of Bangladesh, LGED, Public Private agencies, NGOs	1. Clean river waters as part of environmental sustainability 2. Conservation and maintenance of natural resources, reducing air and water pollution, proper waste disposal and liberating encroached rivers, water bodies, forest areas and khas land

### 3.2 Implementation (gaps) of the existing MSW relevant policies, strategies and action plans

The policy review matrix describes the overall summary of the policies and strategies, with the implementing agencies involved in planning and implementation. It also gives a summarized view of the action plans taken against those existing policies and strategies. (Table-1). Based on these different policies and strategies, several action plans were developed and implemented to some extent for improving the MSW management in Bangladesh. The action plans, their implementation gaps have been discussed elaborately in further sections, triangulating with the qualitative findings and extensive literature review.

Though MSW management of Bangladesh, particularly in Dhaka has improved in the past few years as a result of taking several initiatives by the local authorities – the two city corporations, there are still gaps. One of the most challenging aspects of improving the situation as per the stakeholder's opinion is implementing the proposed strategy mainly due to obstacles in every aspect of execution. The following sections thematically present the current MSW policy scenario, the policy, and implementation gaps (if applicable) from stakeholder and implementing agency perspectives.

#### 3.2.1 Waste generation

The first and key step towards establishing a proper MSW management process is limiting the generation of waste. Almost all the policies and action plans reviewed in this study mention the necessity of reducing the waste generation to reach the sustainable waste management milestone in Bangladesh. Of those, two of the important and active strategies, “National 3R Strategy, 2010” and “Dhaka Structure Plan, 2016” have specifically targeted this issue and formulated action plans accordingly. The 3R strategy encourages reduced use of emission-based technology and private sector investment to promote the “polluters pay” principle. The city corporations and municipalities have taken initiatives for waste reduction through composting based on the 2010 3R strategy action plans. Moreover, the strategy assigns relevant government agencies responsibility to spread public awareness through mass media campaigns and encourage people to reduce waste generation levels starting from the household.

On the other hand, “Dhaka Structure Plan 2016” sets the goal of “Creation of Clean and Pleasant Living Environment” with the objective to ensure the minimization of waste generation in Dhaka city. The strategic plan declares “reduce, reuse and recover” as one of the cornerstones of developing a sustainable MSW management practice in Bangladesh. It emphasizes on reduction of excessive usage of plastic bags in the retail stores by introducing feasible plastic bag reusing practice in the country [21]. The “Poverty Reduction Strategy Paper (PRSP) 2005” also marginally mentioned about probable actions such as promoting and increasing the usage of jute products to reduce the alarming rate of plastic waste generation.

After triangulating the policy and action plan directives with our qualitative findings, we found that significant gaps persist in terms of implementing policies and monitoring policy relevant practices. Study observations reveal that most STSs lack adequate equipment and facilities for effective SWM. The existing capacity of the two Dhaka landfills for managing the generated MSW has been exceeded. Landfill authorities are currently in the process of acquiring additional land for building additional waste disposal platforms in the landfills, which is expected to be completed soon. Our KII stakeholders also mention about practices to reduce waste generation at the source to ensure proper waste management practices rather than steps to keep up with the rising levels of Dhaka's waste generation. KII findings particularly highlight the lack of implementation of existing waste generation and waste segregation policies, which was further substantiated by our co-production workshop findings. Workshop stakeholders categorically emphasized the lack of proper guidelines and information on waste reduction at the household level.

#### 3.2.2 Waste collection and transportation

Waste collection and transportation from the household level to STSs and apparently to the landfills is one of the significant and important parts of MSW management. There are several ordinances, policies and action plans promulgated as part of the sustainable waste collection. In the “Dhaka City Corporation Ordinance of 1983”, it is clearly mentioned that the City corporation is responsible for the removal of waste from all public streets, public latrines, urinals, drains, and all buildings and land under their jurisdiction. It also mentioned that the city corporation is responsible for the supervision of regular waste collection and disposal processes. The “National Strategy for Water Supply and Sanitation, 2014”, emphasized and encouraged the establishment of a community-based primary collection system and link it with the City Corporation's or pourashava's secondary collection, transportation, and final disposal. Under the “National 3R Strategy 2010”, city corporations have constructed/distributed the dustbins in different places. The strategy also mentioned about the transportation of waste from households to the landfills using waste collecting vans, trucks etc. to be provided by the city corporations.

The aspect of source segregation during collection is also equally emphasized on the “National 3R Strategy, 2010” as one of the three targets. The strategy specifically mentioned source segregation during the collection and transportation of waste from households to the landfill sites to make sure the recycling of the refusals. Corroborating the 3R Strategy, the “National Strategy for Water Supply and Sanitation, 2014” also includes a public direction to collect the waste from the streets and roads to ensure the uninterrupted flow of the city drainage system. This strategy also promotes segregation of waste especially while collecting the plastics, poly bags and other recyclable wastes.

However, even though numerous strategies have been formulated with probable action plans for ensuring proper collection and transportation of waste, findings from the observation checklist and KIIs of this study revealed that these are not properly implemented yet. Most of the policies, strategies and action plans specifically mentioned that the waste should be collected on a regular basis from the households; however, the study found that the waste is collected from some households after 2–3 days and the situation is insufficient and even worse in the informal settlements.

The SWM workshop stakeholders also highlighted the limitations in waste collection capacity compared to waste generation levels, an approximate gap of 2000 tons as mentioned by a stakeholder. The uncollected waste is openly dumped in nearby open spaces, increasing the prospect of air, water and soil pollution that has adverse long term public health effects. One of the KII respondents referring to the significance of waste segregation mentioned that even if the waste is segregated before household disposal, the collecting vans dump all the recyclable, non-recyclable, dry and wet waste altogether at the same van, thereby hindering household level implementation of policy practices. During our field work, we also observed that the waste carrying vehicles (vans, trucks, compressors etc.) leak leachate and waste very often while transporting the waste from households to STSs to the landfills, which is a major health and environmental concern. This is also a major gap in the implementation of waste management focused policy.

#### 3.2.3 Waste disposal

The next step towards establishing a proper and sustainable waste management system is appropriate waste disposal following policy and proper disposal guidelines. The aforementioned policies and strategy plans contain specific regulations to be followed by the responsible authorities regarding this. For instance, the “1983 Dhaka City Corporation Ordinance” proclaims that Dhaka City Corporation is responsible for the disposal of all the refuse collected from the public streets, public latrines, urinals, drains, and all buildings and land vested in the Corporation. Although the ordinance does not explicitly define so, disposal of refuse in dustbins or containers and the landfills is widely considered as the responsibility of the City Corporation. They are also responsible for handling the disposal of informal settlements in the city.

However, the study found that the disposal of solid waste is worse in informal settlement areas than in other urban areas. One of the KII respondents mentioned an initiative consisting of the provision of 200 waste bins in informal settlements across Dhaka. Yet, this initiative failed to have long-reaching impacts and there was no further follow-up or monitoring to ensure implementation of proper disposal practices. The field observation findings also revealed the absence of specific waste disposal bins and containers in the informal settlements. It was observed that many dwellers over there dispose of their waste in open spaces within their premises or throw it into a nearby canal. Since the waste collectors do not collect the waste regularly, the openly dumped waste emits a nasty odor in the slum areas. Supporting that, the stakeholders from the co-production workshop also agreed that in the informal settlement areas, the situation is much worse than the other urban residential areas.

On the other hand, “The Bangladesh Environment Conservation Act, 2010” focuses on the mitigation of environmental pollution by controlling the discharge of any environmental pollutants. It addressed restrictions on production, import, storage, loading, and transportation of hazardous wastes in the landfills following “The Environmental Rule, 1997”. Again, the Dhaka structure plan (2016–2035) addressed waste management issues more precisely yet lacks the timely execution of their action plans such as protecting the waterbodies from wastes in Dhaka city. The observation checklist of this study also revealed that there is an alarming rise in river and waterbody pollution due to uncontrolled waste dumping. The Aminbazar landfill, resting on the flood flow zone near the Turag River, has essentially turned into an artificial floating island, as mentioned by one of the KII respondents. The respondent also mentioned that during the summer and rainy season, waste flows with the water from the landfill to the river Turag.

The study finding also revealed that many chemical industries are located in Old Dhaka, and many of these industries dump waste directly into the river Buriganga violating “The Bangladesh Environment Conservation Act, 2010”. From the observation checklist findings, it has been revealed that the local hospitals also dump some of their medical waste such as medical gauze and bandages, empty syringes with attached needles etc. into the STSs which ultimately goes into the landfills and gets mixed with the municipal waste. Waste is even disposed on the roads and public places randomly in some places according to the observation checklist of this study. The stakeholders of the co-production workshop also stated that the uncollected waste on the road causes the water bodies to clog down with waste, consequently blocking the drainage system hindering proper waste management. This clearly reflects a major gap in implementing and monitoring the existing waste disposal policies and strategies in Bangladesh, especially in Dhaka.

#### 3.2.4 Waste recycling and treatment

Waste recycling has become one of the most important priorities in recent times. According to recent estimation, per year approximately 3,23,000 tons of wastes are recycled out of 633,129 tons of plastic waste in Bangladesh [23]. To cope with this increased number, around 6% of the total labor force in Dhaka city is working in the recycling sector. The major recyclable wastes of Dhaka city are plastics, metals/steels, glasses, and papers turning into products as shoes, sandals, boots, buckets, mugs, bottles, media paper, simplex board, glass sheet, steel rods, lamp shade, nuts, bolts, pumps, and cement packaging bags, etc.

Emphasizing this production from recycled wastes, “National Policy for Water Supply and Sanitation, 1998” mentioned about promotion of maximum waste recycling and the use of organic waste materials for compost and biogas production in the private sector. “Private Sector Housing Development Guideline 2005” also proposed waste recycling, composting and biogas generation space in the housing areas. “National 3R Strategy, 2010” encouraged composting and producing bio-gas from organic wastes. Again, “National Urban Sector Policy, 2011” and “Dhaka Environment Management Plan, 2005” emphasized on recycling as a means to reduce solid waste management cost, under the urban environmental management dimension. Several action plans were also undertaken for the implementation of policy directives including “National Environmental Management Action Plan (NEMAP), 1995” which focused on developing garbage disposal and sewerage treatment capability by recycling and economic use of wastes.

This study revealed that recycling industries are mostly run by individuals who buy plastics, papers, or other recycling products from informal vendors or waste collectors. This points to a huge gap in implementing the most recent “National 3R Policy, 2010”. There are several informal factories where the recyclable or non-organic wastes are being used as part of waste into energy generation, but no such treatment or segregation points for the organic waste exists. The organic wastes often reach the landfill without being segregated and converted into compost, bio-gas etc. Composting is done by the private sectors from the waste segregated at the plant. The co-production workshop findings also revealed that as there is no practice of source segregation of waste in Dhaka city, the possibilities like composting biodegradable waste and recycling are not done properly to transform waste into energy. In the case of the waste treatment plants, the stakeholders mentioned that the improper waste treatment methodologies in the landfills are causing harm to the nearby households and environment significantly hence being one of the major barriers to conducting proper waste management practices. The stakeholders opined that the lack of awareness and monitoring in each level of waste management is responsible for this.

#### 3.2.5 Monitoring, regulation, and governance

The information obtained through primary research (qualitative interviews and stakeholders workshop) and secondary literature review both point to a systematic gap in the monitoring of undertaken initiatives, lack of coordination and collaboration among implementing agencies and inability to execute introduced policy actions by respective authorized organizations due to a range of limitations. All these indicate a poor governance structure within the prevailing waste management system in Dhaka.

The KII respondents of our study highlighted the implementation challenges of existing policies as the main barrier to improving the current waste management system. Stakeholders in the workshop also highlighted the lack of coordination and cooperation among executing government agencies. No comprehensive coordination and integration approach exists to address challenges in a systematic manner. “National Environmental Management Action Plan, 2005” described its implementation challenges in a prior report including resource constraints consisting of lack of manpower, budget and organizational capacity of the implementing stakeholders [24].

Another important factor in implementing any policy or action plan is cost and resource management, a sector in which Bangladesh is lagging behind. Financing 3R or 4R strategy-based projects and clean development mechanisms is a challenge for Bangladesh [25]. For instance, one of the most effective strategies for 3R or 4R execution is to raise public awareness towards changing people's behavior and practices towards waste disposal. In this regard, media coverage and campaigns require substantial financing, which is not always accounted for in the budget. The online workshop stakeholders also mentioned that while initiatives are undertaken to change the current behavioral practice, the lack of monitoring and regulation hampers their continuation. An appropriate example might be the lack of monitoring regarding the 200 city corporation provided bins across slums in Dhaka city resulting in a lack of achievable outcome. Additionally, no penalties/fines are imposed regarding the violation of the ordinance.

“The Industrial Policy, 2005” has supported the continuing industrial development occurring in Bangladesh in the last few decades through executing existing laws and other policies. However, there has not been significant or widespread improvement in waste management implementation since the rule was initiated. The reasons included in the industrial policy behind the lack of improvement include low awareness and capacity in the enforcement and inadequate legal provisions, lack of expertise on the issue and resource constraints among others [26]. Moreover, lack of coordination among the implementation agencies, insufficient human resources, budgetary constraints creates barriers in effectively executing the institutional duties and responsibilities [27].

Scarcity of manpower is also evident in municipalities planning and prepare for the implementation of the policies. The study found that, in 2018, out of 329 municipalities in the country, only 27 have graduate and experienced planners whereas in the 2nd and 3rd class municipalities, no planning divisions for the execution of action plans exist, making implementation extremely challenging [28]. Similarly, any development plan requires a vision prior to conceptualization and implementation but in the case of the Dhaka Structure Plan, there is neither a vision nor any implementation strategy [29].

There have been many existing environmental legislation and regulations in Bangladesh to protect the natural resources and environment. However, our findings revealed the environmental rules and legislations are not regularly revised or updated. As mentioned in the qualitative findings, waste spillage from the Aminbazar landfill is polluting the Turag River and its surroundings. Although several projects have been planned and partially executed to protect the river, none of them have been fully implemented due to delayed fund disbursement and inadequate manpower that hampers a strict implementation of waste management and environmental law [30].

In most cases waste management relevant policies included under these legislations are not clearly defined, creating complications for the implementing body, resulting in duplication of tasks and negligence of alternate activities. Hence, even though a policy or action plan may exist on paper, the implementation fails to take effect due to the above-mentioned barriers. A study assumed that the possible reasons for the poor implementation of these environmental acts and policies could be a combination of social, technical, institutional and financial issues [31]. The Government of Bangladesh agreed upon some rules under the “Environmental Conservation Rules (ECR), 1997” for the purpose of determining the standards of air, water, sound, soil and other components of the environment. But the ECR, 1997 has not specified the permissible extent of emissions or the obligations of corrective actions [32]. Additionally, Bangladesh lacks pollution control agreements between local government and industries to control and monitor waste treatment plant operations, waste recycling systems and related experiences at the local level, which would ensure better implementation of these environmental policies [33].

## 4 Discussion

The proper solid waste management system has become a global concern considering the several phenomenal steps from generation to disposal and treatment. For a sustainable MSW management system, waste minimization i.e. waste reduction at source is compulsory. Countries are now focusing on waste minimization such as a study from Brazil found that, waste minimization had considerable environmental impacts particularly decreasing greenhouse gas emissions and, water and soil contamination [34]. Though “National 3R Strategy, 2010” and “Dhaka Structure Plan, 2016” have specifically targeted this issue and formulated action plans accordingly this study found that significant gaps persist in terms of implementing policies and monitoring policy-relevant practices. Awareness programs are yet to become all-inclusive and lack mass propagation, especially at the household level. The absence of reward-punishment incentives and strict law enforcement further undermines the existing waste management policy framework and emerged as a significant gap in the implementation of waste management policy directives. Again, the Republic of Korea is an example of a successful country which has greatly reduced waste generation, even with a large population. They use radiofrequency identification (RFID) chips, entrenched in personal cards that citizens use to open dumpsters and log the weight of the disposed waste [4]. Bangladesh should consider such initiatives to reduce waste at the source.

In the case of the waste collection system, this study finding revealed that in many parts of Dhaka city, especially in the informal settlements, MSW is not collected regularly, whereas in cities like Singapore city; the capital of Hong Kong, Beijing; Tokyo; and Seoul collect almost 100 percent of generated waste. Singapore follows the “Clean Land Policy” to reduce and recycle the waste as much as possible and minimize the volume of waste disposed of [35]. Japan also adopts advanced technology to collect 100% waste considering sustainable waste management [36]. In addition to the east Asian developed countries, some South Asian cities such as Pune also collect 100% solid waste [4]. To achieve 100% waste collection rate, the Pune city of India follows a public-private partnership with the organization named Solid Waste Collection and Handling (SWaCH), which is also officially known as SWaCH Seva Sahakari Sanstha Maryadit. This public-private partnership model was based on the recovery of user fees from the waste management service users as well as the provision of infrastructure and management support from the Municipal Corporation of Pune [37].

To improve the MSW collection system, stakeholders from the workshop also emphasized how the integration of GIS (Geographic Information System) in the existing waste management system can improve the current waste framework. A study conducted in the municipality of Sfax, Tunisia shows that an optimized route with a change of collection mode allows savings of about 40%, 57%, 40.5%, and 48% in the number of workers, working time, traveled distance, and fuel consumption, respectively, and hence a gain of about 60,000 TND/year, in addition to other benefits related to CO2 emissions, hours of work, vehicles wear/maintenance, and so forth compared to previous scenario [38]. Thus the use of GPS and GIS, or even route optimization software, may be considered to improve the current collection coverage [39]. In New York City, United States, a pneumatic waste collection system has been set up underground, whereby all buildings are connected to the central point of treatment and disposal through a tube or via vacuum [40]. This pneumonic system is a healthier alternative a truck-based collection systems, and further reduces transportation and infrastructure cost. Bangladesh may consider a similar initiative to triumph the SWM system [35, 41].

Though waste segregation is mandatory in most of the developed countries this study did not observe any formal waste segregation process throughout our study. Germany can be considered an example to follow regarding waste separation. In Germany, it is compulsory by law for waste producers to sort the waste in separate bins to reuse and compost [42]. Again Milan, Italy is the first metropolis with extensive Source Segregation Organic (SSO) collection [39]. A European Environment Agency reports stated that countries with successful waste management systems promote incineration against recycling in their national policies [43, 44]. Waste separation at the source should be compulsory and should be considered as a regulatory policy instrument that may contribute to economic and efficient recycling [44]. Again, enforcement of strict laws regarding waste segregation similar to the above-mentioned countries may help to reduce the level of final waste disposal with far-reaching environmental benefits.

Even though many policies emphasized waste recycling and treatment along with several action plans developed for implementation, our study findings revealed that formal waste recycling is not prioritized in Bangladesh even though it is widely argued that recycling helps to ensure environmental sustainability by substituting raw material inputs into and redirecting waste outputs out of the economic system [45]. Hence, EU waste policies have developed significantly over the last 30 years through some Environmental Action Plans (EAPs) and a framework of waste law focusing on the elimination of landfilling, limitation of incineration to non-recyclable materials, and to the absolute decline of waste generated per capita [46]. In response to the emerging “circular economy” trend, countries now largely focus on waste recycling in their policy, such as Japan's sound material-cycle society [47], the European Union's recycling society and circular economy, etc. [48]. Many EU countries have laws for organic waste recycling in the early stage of the national implementation of bio-waste management thus many of them already recycled more than 50% of their waste [4]. But in South Asian countries including Bangladesh, the rate is still low due to a lack of proper policy execution also the recycling sectors are rarely monitored by government agencies. Furthermore, waste management-related policies are not even revised timely in Bangladesh.

Overall, the challenges and gaps in the implementation of waste management-related policies are quite similar in most cases. Lack of coordination, resource and budget constraints, lack of manpower and knowledge among the general people and implementing bodies are the major concerns that need to be taken care of, for public health and environmental wellbeing. The ADB 2003 report suggests proper coordination among the agencies is necessary to strengthen the institutional system and subsequently improve the operational efficiency of any policy or strategic plan [49]. Many national strategies and action plans also emphasize executing Public-Private Partnership (PPP) for proper solid waste management. However, a study suggests that the PPP project implementation capability, especially the regulatory framework, transaction expertise, and PPP financing facilities have not improved over the last decade, leaving a major gap in the implementation of the policies [50]. By overcoming the challenges and fulfilling the gaps of proper implementation of the existing waste management laws and policies, it is possible to aim for a cleaner and healthier Dhaka city.

## 5 Study recommendations

Based on the research findings this study recommends some initiatives to ensure a sustainable waste management system in the country, especially in Dhaka city. These recommendations have been generated through triangulating the findings from the observations, KIIs and co-production workshop on solid waste management in Dhaka and presented categorically in the following sections.

### 5.1 Waste generation, collection and disposal

In order to reduce waste generation, this study recommends to implementing a community-led total sanitation approach for waste generation at the household level. It is suggested to ensure making particular laws regarding waste collection on a regular basis, and mandatory enforcement of those for the uncollected wastes. Besides, regular collection from the informal settlements by introducing subsidized waste collection fees and provisions should be mentioned in those laws or policies. Moreover, there should be a common collection point in the informal areas and the need to encourage the dwellers to put their waste in those specified points. It is also recommended to strictly monitor whether they can segregate and discharge the waste properly in those points.

### 5.2 Waste recycling and treatment

The stakeholders of the co-production workshop recommended that segregation should be started from the household level as dumping site hazards are increasing due to the lack of source segregation, especially at the households. All the kitchen waste and plastic waste should be separated at the households and a weekly scheduled collection system can be introduced in the community. For example, organic kitchen waste should be collected each day, non-degradable recycled waste can be collected 4–5 days per week. For the source segregation, recycling and reusing practice, it can be effective to introduce a system of providing incentives to the common citizens by the government. For instance, giving some money or rewards for each empty plastic bottle or any other plastic/recyclable product. In the community level and STSs, the authority should manage separate bins according to compostable and non-compostable wastes. The key informants recommended having a proper plan of using waste as a resource and suggesting generate fuel (gas) from landfill organic waste. Besides, urban agriculture such as rooftop gardening can be a way to manage waste regarding compost as per their suggestion.

### 5.3 Awareness, regulations and managements

To cope with the overall management in each step of waste management, it is recommended to develop specific policies regarding the awareness campaigns to guide the behavior of the general population. Waste segregation in the household to landfill level should be emphasized on those campaigns. The policies need to mention about the specific distribution of the financial and human resources managements assigning a regulatory body to maintain this. Investment and strict monitoring in collaboration, communication, dialogue, education, and information is necessary to implement the existing policies. Furthermore, it is mandatory to implement strict laws on deciding how this should work and how the specific areas of work should be decided among the main implementing agencies. It is recommended to increase the budget regarding the safety equipment and specify the health and safety concerns of the waste handlers in the policies. The government can take initiatives to introduce health insurance for the waste workers and also mandate their education of them about the use of safety equipment. A reward-punishment system for the citizens in each step of waste management should be considered in policies, the stakeholders opine this as an effective way to encourage them. Public-private partnership to connect the different sectors of waste management is highly recommended while developing such policy, strategy or action plans. Lastly, there should be a unique institution or authority to manage the whole procedure and direct all the authorized institutions involved in waste management, i.e. from waste generation to waste disposal who will bring together all the authorities directly related to solid waste management.

## 6 Limitations & challenges of the study

This study has several limitations that need to be acknowledged. One of the major challenges of this study was collecting the updated policy documents from the online for the comprehensive review of the policies and strategies. Few of these documents were collected as hardcopies and needed translations from Bangla to English which apparently made this study a bit lengthy process. Moreover, the result of this study comprises a lot of components of the waste management system in Dhaka city which made it less focused on any specific aspect of the research topic. To fulfill this gap and cope with the global waste management inventiveness, this study recommends that future research to be more focused on the 3R strategy embedded in the waste management field of the country.

## 7 Conclusion

Waste management is a cross-cutting sector impacting various aspects of the environment, society, and the economy. Poor waste management has strong linkages to a range of other challenges such as adverse health outcomes, climate change, soil contamination, air and water pollution, and loss of animal and marine life. In general, the policies and legislation in place for solid waste management and other effluent are well constructed and comprehensive. The 3R strategy, Dhaka Structure Plan, and Seventh Five-Year Plan (FY2016-2020) all include adequate provisions for solid waste management. However, there are few action plans in place that lack of skills and expertise to ensure that both government and private sector developments adequately address solid waste management concerns. With a few exceptions, there is still a lack of institutional coordination and awareness, let alone the capacity to address policy goals and objectives. Hence, waste management should be considered a priority area for sustainable development, with policymakers formulating feasible and contextualized policies for the country following the successful neighboring developing countries.

## Declarations

### Author contribution statement

Delufa Tuz Jerin and Hasna Hena Sara: Performed the experiments; Analyzed and interpreted the data; Contributed reagents, materials, analysis tools or data; Wrote the paper.

Marzuka Ahmad Radia, Prianka Sultana Hema, Shahriar Hasan and Salma Akter Urme: Contributed reagents, materials, analysis tools or data.

Camilla Audia: Analyzed and interpreted the data; Contributed reagents, materials, analysis tools or data.

Tanvir Hasan and Zahidul Quayyum: Conceived and designed the experiments; Contributed reagents, materials, analysis tools or data; Wrote the paper.

### Funding statement

This work is supported by the Pathways to Equitable Healthy Cities grant from the Wellcome Trust [209376/Z/17/Z].

### Data availability statement

Data will be made available on request.

### Declaration of interests statement

The authors declare no conflict of interest.

### Additional information

No additional information is available for this paper.

## Footnotes

2 Municipal solid waste (MSW) is a pool of various solid wastes by towns and cities from different types of household activities. Source: https://www.sciencedirect.com/science/article/pii/B978012803837600007X.
